# IMPDH2 and HPRT expression and a prognostic significance in preoperative and postoperative patients with osteosarcoma

**DOI:** 10.1038/s41598-021-90456-4

**Published:** 2021-05-25

**Authors:** Parunya Chaiyawat, Areerak Phanphaisarn, Nutnicha Sirikaew, Jeerawan Klangjorhor, Viraporn Thepbundit, Pimpisa Teeyakasem, Phichayut Phinyo, Dumnoensun Pruksakorn, Jongkolnee Settakorn

**Affiliations:** 1 Musculoskeletal Science and Translational Research (MSTR) Center, Faculty of Medicine, Chiang Mai University, Chiang Mai, Thailand; 2 Center of Multidisciplinary Technology for Advanced Medicine (CMUTEAM), Faculty of Medicine, Chiang Mai University, Chiang Mai, Thailand; 3 Department of Family Medicine, Faculty of Medicine, Chiang Mai University, Chiang Mai, Thailand; 4 Center for Clinical Epidemiology and Clinical Statistics, Faculty of Medicine, Chiang Mai University, Chiang Mai, Thailand; 5 Department of Orthopedics, Faculty of Medicine, Chiang Mai University, Chiang Mai, Thailand; 6 Department of Pathology, Faculty of Medicine, Chiang Mai University, Chiang Mai, Thailand

**Keywords:** Bone cancer, Prognostic markers, Cancer metabolism, Sarcoma, Cancer therapy

## Abstract

Osteosarcoma is one of the most aggressive bone tumors in children and adolescents. Development of effective therapeutic options is still lacking due to the complexity of the genomic background. In previous work, we applied a proteomics-guided drug repurposing to explore potential treatments for osteosarcoma. Our follow-up study revealed an FDA-approved immunosuppressant drug, mycophenolate mofetil (MMF) targeting inosine-5′-phosphate dehydrogenase (IMPDH) enzymes, has an anti-tumor effect that appeared promising for further investigation and clinical trials. Profiling of IMPDH2 and hypoxanthine–guanine phosphoribosyltransferase (HPRT), key purine-metabolizing enzymes, could deepen understanding of the importance of purine metabolism in osteosarcoma and provide evidence for expanded use of MMF in the clinic. In the present study, we investigated levels of IMPDH2, and HPRT in biopsy of 127 cases and post-chemotherapy tissues in 20 cases of high-grade osteosarcoma patients using immunohistochemical (IHC) analysis. Cox regression analyses were performed to determine prognostic significance of all enzymes. The results indicated that low levels of HPRT were significantly associated with a high Enneking stage (*P* = 0.023) and metastatic status (*P* = 0.024). Univariate and multivariate analyses revealed that patients with low HPRT expression have shorter overall survival times [HR 1.70 (1.01–2.84), *P* = 0.044]. Furthermore, high IMPDH2/HPRT ratios were similarly associated with shorter overall survival times [HR 1.67 (1.02–2.72), *P* = 0.039]. Levels of the enzymes were also examined in post-chemotherapy tissues. The results showed that high IMPDH2 expression was associated with shorter metastasis-free survival [HR 7.42 (1.22–45.06), *P* = 0.030]. These results suggest a prognostic value of expression patterns of purine-metabolizing enzymes for the pre- and post-chemotherapy period of osteosarcoma treatment.

## Introduction

Osteosarcoma is a malignant bone cancer that is more common in children and adolescents. Currently standard treatment includes a combination of chemotherapy and surgery. Since the introduction of chemotherapy in combination with surgery in 1970, the survival rate of osteosarcoma cancer patients with localized disease has increased to 70%^1,2^. However, the five-year survival rate of patients not responding to chemotherapy is remains 30%^3,4^. Our previous study reported 37.9% of the 5-year overall survival rates in Northern Thailand^5^. This finding is comparable with studies conducted within Asia–Pacific region reporting 27–48% of the 5-year overall survival rates that were much lower in comparison to western regions with rates between 60 and 70%^6^. Major factors of the poor clinical outcomes were presence of metastasis and not completing treatment regarding patients’ non-compliance to treatment regime.

The main reason treatment for osteosarcoma has not improved the survival rate over the past 30 years is due to an inability to specify the key mechanisms involve in the occurrence and spread of osteosarcoma. Information obtained from whole genome and exome sequencing has indicated that osteosarcoma has a highly chaotic genetic background including chromosome aberrations, chromothripsis, kataegis, and genome instability with a high rate of copy number alterations (CNAs)^7,8^. There is also evidence of epigenetic changes resulting from histone modifications and DNA methylation^9^. These abnormalities result in changes in protein levels in both oncogenic proteins and tumor suppressors.

Cancer cells divide rapidly with a significantly higher rate of DNA and RNA replication. Cell division is controlled by many types of transcription factors and involves changes in volume, energy, and anabolic metabolism to pass through the S-phase in the cell cycle^10^. Cancer cells require both energy metabolism and increased nucleotide biosynthetic pathways. Purine nucleotides are the most important source of cellular energy and are involved in diverse signaling pathways^11^. Biosynthesis of purine nucleotides occur via two mechanisms, the “salvage pathway” that reuses biomolecules derived from DNA and RNA degradation and the “de novo pathway”, synthesis of purines from small molecules derived from cellular biochemical processes^12,13^. The de novo pathway produces greater amounts of purine nucleotides than the salvage pathway, but it uses more energy and involves more complex reactions^14^. It has been found that purine nucleotides are produced under many conditions, mainly through the de novo pathway. The most obvious example is the activation of the de novo pathway in active B and T lymphocytes^15^, as well as in various types of cancer^16^. Nucleotide metabolism in cancer cells has been studied for several decades. In 1975, a study by Jackson et al. first demonstrated the importance of the de novo pathway in nucleotide synthesis in liver cancer cells^17^. They reported higher activity of glutamine PRPP amidotransferase enzyme (GPAT; EC 2.4.2.14), an enzyme regulating synthesis of inosine monophosphate (IMP) in liver cancer cells, with significantly lower IMP degradation. They also found increasing activity of inosine-5′-phosphate (IMP) dehydrogenase (IMPDH), the enzyme with the highest activity level in the fastest dividing liver cancer cells tested in the experiment. IMPDH is an enzyme that is important in the process of guanine nucleotide synthesis through the de novo pathway. It accelerates the conversion of IMP to xanthosine 5′-phosphate (XMP), the rate-limiting step in the guanine nucleotide synthesis process. Proliferation of cancer cells depends largely on the amount of nucleotides within the cell as well as the activity of key enzymes regulating the rate-limiting step of the nucleotide synthesis process. Furthermore, cancer cells strongly rely on the glycolysis and the TCA cycle to obtain enough ribose-5-phosphate, a primary precursor of de novo nucleotide synthesis, to boost their intracellular nucleotide content. Ribose-5-phosphate is synthesized from the pentose phosphate pathway (PPP) through the nonoxidative branch, in which cancer cells vigorously stimulate the PPP through glycolytic flux by oncogenic signaling of the PI3K-Akt pathway^18^. Taken together, this makes the de novo purine nucleotide synthesis pathway an attractive target for cancer treatment.

Inosine-5′-phosphate (IMP) dehydrogenase (IMPDH) is a well-known target for antileukemic and immunosuppressive therapy^19^. Inhibiting IMPDH results in decreased cellular guanine nucleotide pools and subsequently modulates the synthesis of DNA and RNA which is lethal for cells. Human IMPDH consists of 2 isoforms, IMPDH1 and IMPDH2, that are encoded from different genes. Both IMPDH isoforms share a similar amino acid sequence (84%), structure (95%), kinetic properties, and substrate binding site^20^. Expression of IMPDH2 is enhanced significantly in rapidly proliferating cells including various types of cancers including colorectal, bladder, prostate, kidney, nasopharyngeal carcinoma and leukemia^21–24^. Decades ago, researchers explored a link between Myc oncogene and production of nucleotides to support a high growth rate of cancer cells. Oncogenic signaling through the Myc pathway directly controls glutamine uptake and increases expression of various rate-limiting enzymes in de novo nucleotide synthesis including, but not limited to, *IMPDH1* and *IMPDH2* genes^14,25^.

Hypoxanthine guanine phosphoribosyltransferase (HPRT) is a key enzyme in salvage pathway responsible for the formation of GMP and IMP by recycling guanine and inosine from degraded DNA and RNA, respectively^26^. To maintain cellular GTP level in somatic cells, HPRT is reliably expressed in low level^27^. Unlike in normal cells, recent study showed that HPRT expression is highly variable in various cancer tissues including lung, breast, colon, prostate and pancreas^27^. HPRT activity was also detected in human osteosarcoma xenografts, in which the activity ranging from 0.97 to 4.06 nmol/min/mg of protein at pH 7.4^28^. Further study of human osteosarcoma tissues showed that all samples contained substantial activities of HPRT enzyme within the range of xenografts of human osteosarcoma^29^.

Mycophenolate mofetil (MMF) is an immunosuppressive agent approved for use in patients receiving allogeneic renal, cardiac or hepatic transplants to prevent organ rejection^30^. MMF is a prodrug of mycophenolic acid (MPA), an inhibitor of IMPDH^31^. The main reason MPA is a potent immunosuppressive agent is that activated T- and B-lymphocytes are highly divided based on de novo purine nucleotide synthesis^30^. A previous study by the authors unveiled the potency of MPA, a pan-IMPDH inhibitor, as an anti-tumor agent for osteosarcoma^32^. That study showed MPA effectively inhibits osteosarcoma cell viability and also reduces tumor mass in animal models at clinically achievable doses^32^. The study also demonstrated an effect of MPA in the modulation of pulmonary metastasis of osteosarcoma.

To enhance understanding of guanine nucleotide synthesis at population levels, this longitudinal study investigated expression patterns of individual key enzymes regulating de novo and salvage guanine nucleotide pathways in osteosarcoma samples. Furthermore, accumulating studies have reported that cancer cells have an increase in the activation of nucleotide anabolism and a lower rate of nucleotide catabolism^14^. Integrative analysis of IMPDH2 and HPRT will provide valuable data on an association of rewired GTP synthesis system in osteosarcoma and clinical outcomes of the patients.

## Methods

### Patient characteristics

The present study recruited 127 osteosarcoma patients who had been diagnosed and treated at Maharaj Nakorn Chiang Mai Hospital, Thailand, between 1999 and 2018. Patients were followed-up for survival and metastasis-free survival until 11 January 2020. Clinicopathological parameters, including date of diagnosis, date of metastasis, Enneking staging, location of tumor, and metastatic status were retrieved from hospital records and pathology reports (Table 1). All primary biopsy and post-operative chemotherapy tissue slides were evaluated histologically by a bone and soft tissue pathologist (JS). Inclusion criteria for post-operative chemotherapy studies included post-operative necrosis report available and percentage of tumor necrosis ≤ 80%.

**Table 1:** Characteristics of osteosarcoma patients in study cohort.

Factor	All patients	IMPDH2 expression			HPRT expression		
		Low (IRS ≤ 8)	High (IRS > 8)	*P* value	Low (IRS ≤ 2)	High (IRS > 2)	*P* value
**Age at diagnosis (years)**							
≤ 15	65	37 (57%)	28 (43%)	> 0.999	19 (29%)	46 (71%)	0.220
> 15	62	36 (58%)	26 (42%)		12 (19%)	50 (81%)	
**Gender**							
Male	68	35 (52%)	33 (48%)	0.154	18 (26%)	50 (74%)	0.680
Female	59	38 (64%)	21 (36%)		13 (22%)	46 (78%)	
**Enneking stage**							
IIB	72	41 (57%)	31 (43%)	0.545	13 (18%)	59 (82%)	**0.023**
III	39	25 (64%)	14 (36%)		15 (38%)	24 (62%)	
**Site**							
Extremities	104	61 (59%)	43 (41%)	> 0.999	14 (33%)	80 (77%)	0.750
Axial	15	9 (60%)	6 (40%)		4 (27%)	11 (73%)	
**Metastasis**							
No	36	21 (58%)	15 (42%)	0.742*	4 (11%)	32 (89%)	0.024*
At follow-up	36	20 (56%)	16 (44%)		9 (25%)	27 (75%)	
At diagnosis	39	25 (64%)	14 (36%)		15 (38%)	24 (62%)	

*P* values were calculated with Fisher’s exact test.

*P values were calculated with Chi-square test.

This study protocol has been approved by the Research Ethics Committee of the Faculty of Medicine, Chiang Mai University. All patients and/or parent provided informed consent for patient information to be stored in the hospital database. All methods were carried out in accordance with good clinical practices (GCPs) and relevant guidelines.

### Immunohistochemistry and scoring

Immunohistochemistry was performed on formalin-fixed paraffin-embedded (FFPE) tissues from archival biopsy and post-chemotherapy paraffin blocks at the Department of Pathology, Faculty of Medicine, Chiang Mai University. We applied the Ventana automated staining system and an Ultraview Universal DAB Detection Kit (both from Ventana Medical Systems, Tucson, AZ, USA) to detect primary antibodies including anti-IMPDH2 (dilution 1:500, Abcam Cat# ab131158, RRID:AB_11156264) and anti-HPRT (dilution 1:300, Abcam Cat# ab10479, RRID:AB_297217) (all from Abcam, Cambridge, UK). Cytoplasmic staining of IMPDH and HPRT was independently evaluated by two observers (by PC and JS), blind to patient outcome data, and scored by using a semi-quantitative immunoreactive scoring system^9^. The percentage of immunoreactive cells was estimated and scored as follows: negative = 0, positive staining < 10% = 1, positive staining ≥ 10 and < 33% = 2, positive staining ≥ 33% and < 66% = 3, positive staining ≥ 66% = 4. Intensity of staining was scored on a scale of 0–3: no color reaction = 0, weak staining = 1, moderate staining = 2, and strong staining = 3. Immunoreactive score (IRS) was derived by multiplying immunoreactive cell scores and intensity of staining scores to compute an immunoreactive score ranging from 0 to 12 defined as negative; IRS = 0, weak; IRS > 0–4, moderate; IRS > 4–8, and strong; IRS > 8–12.

### Statistical analysis

Statistical analysis was performed using STATA version 11 (Stata, RRID:SCR_012763) and GraphPad Prism version 8.4.2 (GraphPad Prism, RRID:SCR_002798) (GraphPad Software, Inc., La Jolla, CA, USA). The statistical significance of correlations between expressions of IMPDH and HPRT and clinicopathologic data were determined by Fisher’s exact and Chi-square test. Receiver operating characteristic (ROC) curve analysis was applied to define the optimal cutoff points for IMPDH2 and HPRT expression levels as well as IMPDH2/HPRT ratio. Associations of IMPDH, HPRT and IMPDH2/HPRT ratio with overall survival and metastasis-free survival of osteosarcoma patients were evaluated by the Kaplan–Meier method with the log-rank test. Multivariate survival analysis was determined using Cox regression of proportional hazards to probe for significance at the 95% confidence interval (CI). *P* values < 0.05 were considered to be statistically significant.

## Results

### Expression patterns of IMPDH2 and HPRT in the osteosarcoma cohort

**Figure 1:**
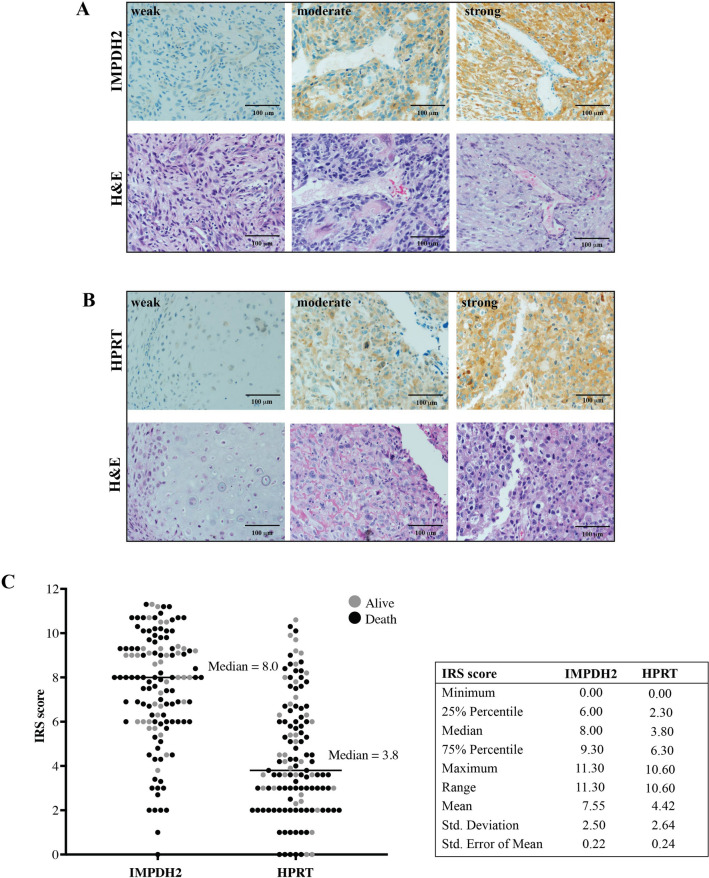
Immunohistochemical staining of (**A**) IMPDH2 and (**B**) HPRT in osteosarcoma tissues (Χ400). (**C**) Distribution of IMPDH2 and HPRT levels in osteosarcoma cohort (N = 127). H&E; hematoxylin and eosin.

To examine expression profiles and staining patterns of IMPDH2 and HPRT enzymes, we conducted immunohistochemical analysis of formalin-fixed paraffin-embedded tissues from biopsy samples of 127 osteosarcoma cases and 20 post-chemotherapy samples. We found IMPDH2 and HPRT expressed mainly in the cytosol of the osteosarcoma cells (Fig. 1A, B). The majority were positive for IMPDH2 (99%) and HPRT (94%) staining. Interestingly, we observed a reverse overall distribution between IMPDH isoforms and HPRT staining patterns. We found that the 42% of patients had strong IMPDH2 staining, whereas only 11% had strong HPRT staining (IRS > 8). Expression patterns of IMPDH2 and HPRT stanning are shown in Fig. 1C.

### Correlations of IMPDH2 and HPRT staining patterns with clinicopathological data

Characteristics of 127 osteosarcoma patients in the cohort are shown in Table 1. By comparing between expression levels of IMPDH2 and HPRT in biopsy tissues and individual primary characteristics of patients, we found a negative correlation between HPRT levels with Enneking stage (*P* = 0.023) as well as metastatic status (*P* = 0.024).

### Survival analysis of IMPDH2 and HPRT in osteosarcoma

In the survival analysis, we excluded data from 8 osteosarcoma patients who were lost to follow-up. Overall, survival time of 119 patients ranged from 1 to 209 months after initial diagnosis (median = 18 months). Seventy percent (84 cases) of the patients died after 1 to 102 months (median = 13 months). The 1-year and 5-year survival rates were 70% and 32%, respectively.

**Figure 2:**
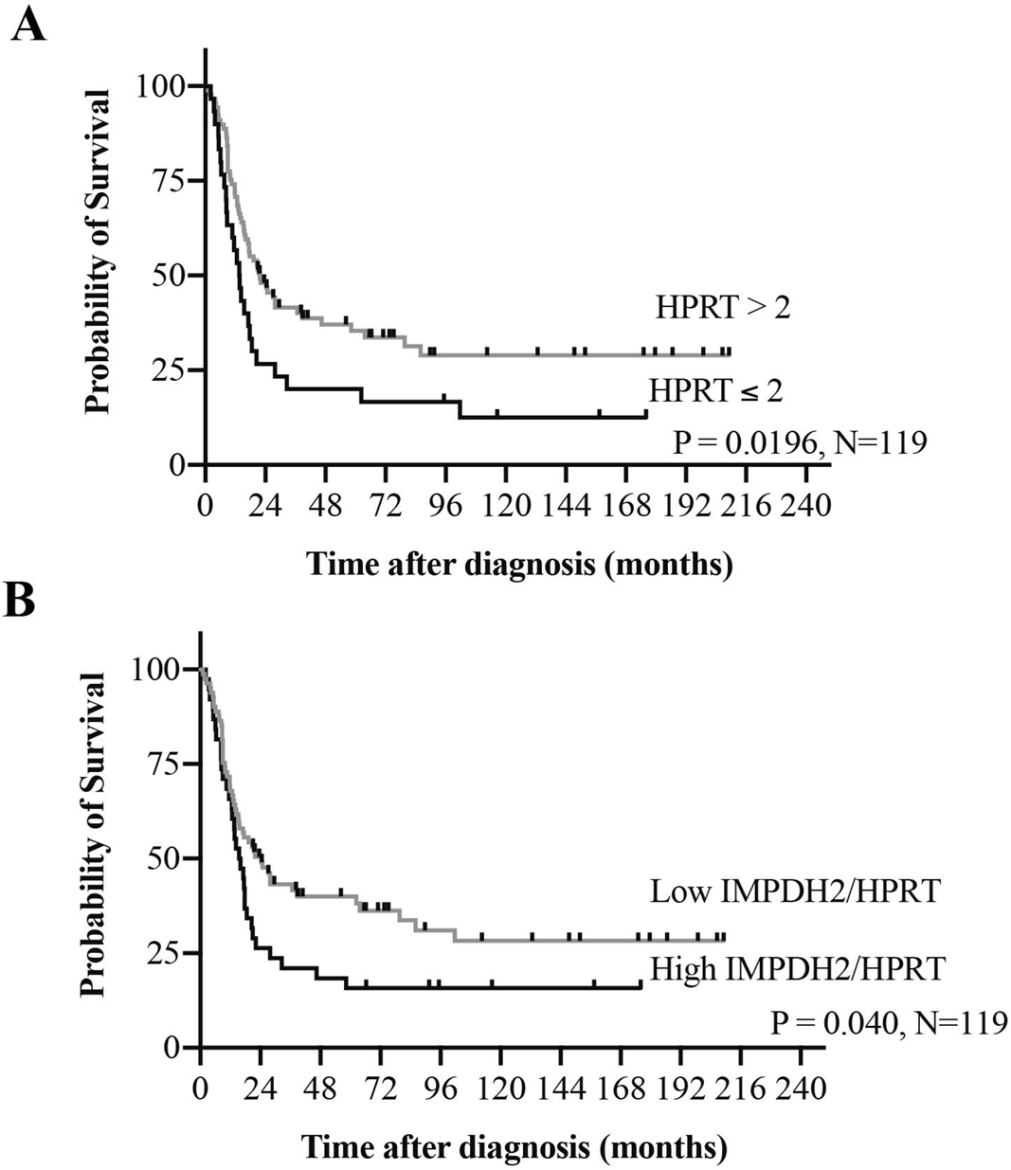
Kaplan–Meier curves showing overall survival by (**A**) HPRT and (**B**) IMPDH2/HPRT ratio in osteosarcoma cases.

Immunoreactive score (IRS) cutoff points for each of the purine metabolizing enzymes were defined by receiver operating characteristic (ROC) curves: IMPDH2, IRS = 8; HPRT, IRS = 2. Then, univariate survival analysis of individual enzymes (IMPDH2 and HPRT) was conducted using Kaplan–Meier survival analysis and log rank tests. We found that low expression levels of HPRT were associated with shorter overall survival times (*P* = 0.0196), but not for IMPDH2 results (Fig. 2A; Tables 2, 3). The median survival time of patients with low HPRT was 13.6 months, whereas patients with high HPRT expression had a median survival of 21.9 months. Multivariate survival analysis showed that low expression of HPRT [HR 1.70 (1.01–2.84), *P* = 0.044] was an independent prognostic factor for low survival time (Table 4). Other significant prognostic factors for low survival time in this cohort included advanced Enneking stage (*P* = 0.001), not completing treatment (*P* < 0.0001) and the presence of metastasis (*P* < 0.0001).

**Table 2:** Patient survival analysis (N = 119).

Factor	Patients	Events (death)	Median survival (months)	*P* value (Log rank test)
**Age at diagnosis (years)**				
≤ 15	58	40	20.5	0.384
> 15	61	44	17.0	
**Gender**				
Male	64	50	17.7	0.258
Female	55	34	20.5	
**Enneking stage**				
IIB	71	42	27.1	**0.001**
III	38	32	12.5	
**Site**				
Extremities	102	70	20.6	0.183
Axial	13	10	12.7	
**Metastasis**				
No	36	12	Undefined	**< 0.0001**
Yes	73	62	15.4	
**Treatment**				
Completed treatment	74	44	27.8	**< 0.0001**
Not completed treatment	45	40	9.0	
**IMPDH2 expression**				
Low (IRS ≤ 8)	70	53	15.5	0.119
High (IRS > 8)	49	31	21.4	
**HPRT expression**				
Low (IRS ≤ 2)	30	26	13.6	**0.0196**
High (IRS > 2)	89	58	21.9	
**IMPDH2/HPRT ratio**				
Low IMPDH2/HPRT	81	52	24.4	**0.040**
High IMPDH2/HPRT	38	32	15.8	

*P* values were calculated using the log-rank test. *P* values < 0.05 shown in bold.

**Table 3:** Multivariate analysis of factors associated with overall survival in the osteosarcoma cohort with inclusion of IMPDH2 expression.

Factor	Overall survival	
	HR (95% CI)	*P *value
**Age at diagnosis (years)**		
≤ 15	1.00	–
> 15	1.30 (0.80–2.11)	0.289
**Site**		
Extremities	1.00	–
Axial	1.04 (0.49–2.20)	0.915
**Stage**		
IIB	1.00	–
III	2.43 (1.48–3.99)	**< 0.001**
**Treatment completion**		
Completed	1.00	–
Not completed	3.10 (1.89–5.08)	**< 0.001**
**IMPDH2 expression**		
Low (IRS ≤ 8)	1.00	–
High (IRS > 8)	1.40 (0.87–2.27)	0.169

**Table 4:** Multivariate analysis of factors associated with overall survival in the osteosarcoma cohort with inclusion of HPRT expression.

Factor	Overall survival	
	HR (95% CI)	*P *value
**Age at diagnosis (years)**		
≤ 15	1.00	–
> 15	1.30 (0.80–2.12)	0.286
**Site**		
Extremities	1.00	–
Axial	1.14 (0.53–2.48)	0.736
**Stage**		
IIB	1.00	–
III	2.38 (1.44–3.91)	**0.001**
**Treatment completion**		
Completed	1.00	–
Not completed	3.20 (1.94–5.27)	** < 0.001**
**HPRT expression**		
High (IRS > 2)	1.00	–
Low (IRS ≤ 2)	1.70 (1.01–2.84)	**0.044**

Following up on the significant association between low HPRT and poor prognosis, we further analyzed our cohort by considering the combinational effect of IMPDH2 and HPRT enzymes. The results indicated a remarkable strong correlation between high IMPDH2/HPRT ratios and shorter survival time (Fig. 2B). Median survival of patients with a high IMPDH2/HPRT ratio was 15.8 months, whereas patients with a low IMPDH2/HPRT had a median survival of 24.4 months. The multivariate survival analysis results showed that a high IMPDH2/HPRT ratio [HR 1.67 (1.02–2.72), *P* = 0.039] was an independent poor prognosis factor (Table 5).

**Table 5:** Multivariate analysis of factors associated with overall survival in osteosarcoma with inclusion of IMPDH2/HPRT ratio.

Factor	Overall survival	
	HR (95% CI)	*P *value
**Age at diagnosis (years)**		
≤ 15	1.00	–
> 15	1.36 (0.83–2.23)	0.216
**Site**		
Extremities	1.00	–
Axial	0.92 (0.43–1.97)	0.841
**Stage**		
IIB	1.00	–
III	2.38 (1.45–3.91)	**0.001**
**Treatment completion**		
Completed	1.00	–
Not completed	3.03 (1.85–4.97)	**< 0.001**
**IMPDH2/HPRT ratio**		
Low	1.00	–
High	1.67 (1.02–2.72)	**0.039**

## Metastasis-free survival analysis of IMPDH2 and HPRT expression in post-chemotherapy tissues

**Figure 3:**
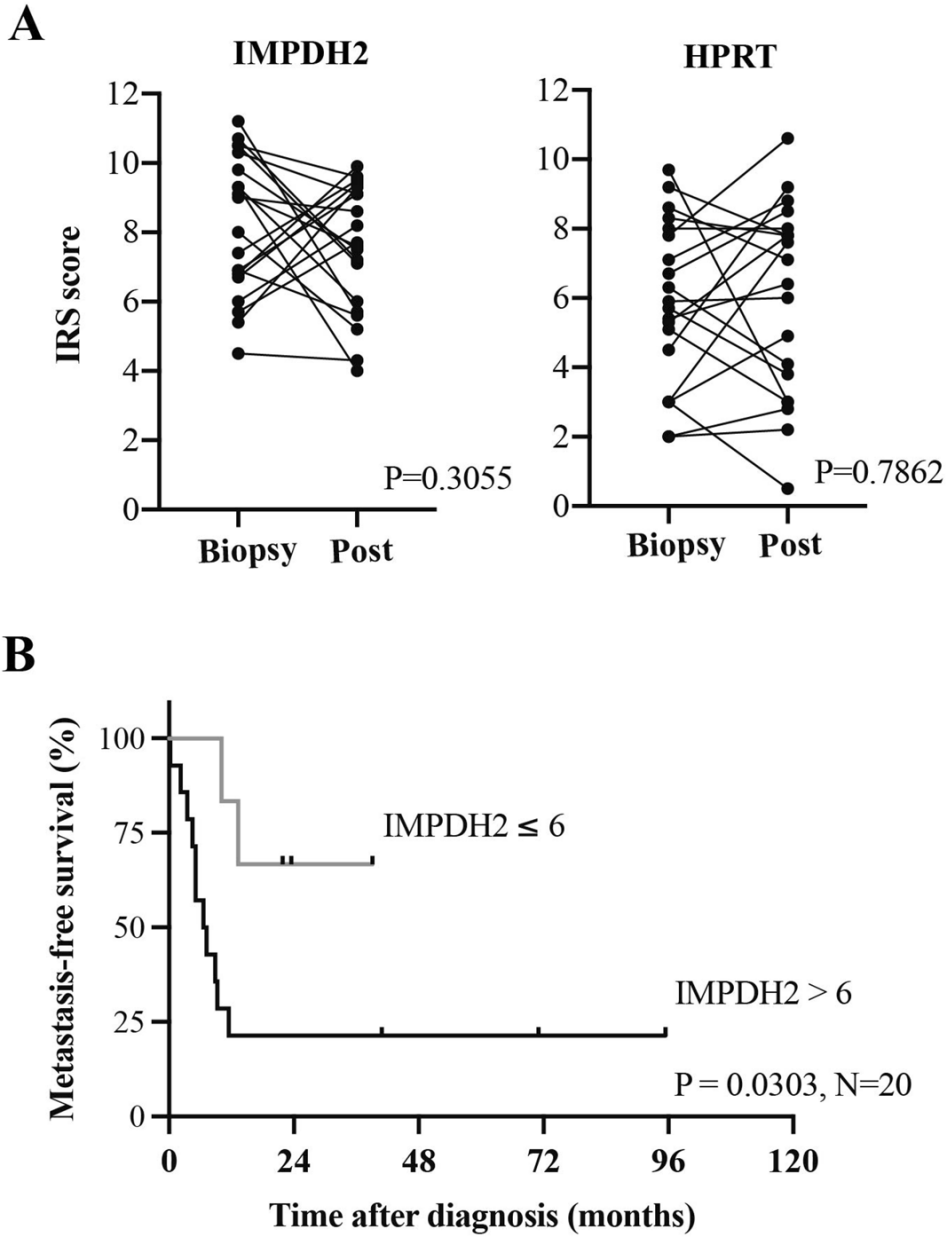
Expression levels of IMPDH2 and HPRT enzymes in longitudinal samples. (**A**) Expression of IMPDH2 and HPRT in biopsy and post-chemotherapy tissues. (**B**) Metastasis-free survival analysis of IMPDH2 in post-chemotherapy tissues of osteosarcoma.

In this study, we assessed expression of IMPDH2 and HPRT enzymes in biopsies as well as in post-operative chemotherapy tissues of 20 osteosarcoma patients. The results demonstrated that expression of IMPDH2 and HPRT was maintained at the same level in post-operative chemotherapy tissues as in biopsy samples (Fig. 3A). We further analyzed the association between the levels of each of the three enzymes and metastasis-free survival. Univariate and multivariate analysis results showed that high levels of IMPDH2 (IRS > 6) in post-chemotherapy tissues was an indicator of a poor prognosis [HR 7.42 (1.22–45.06), *P* = 0.030] (Fig. 3B; Tables 6, 7), whereas no association between HPRT level or IMPDH2/HPRT ratio and metastasis-free survival was observed in post-chemotherapy tissues (Tables 6, 7, 8, 9). Other significant prognostic factors for short metastasis-free period in this cohort included female gender (*P* = 0.027) and advanced Enneking stage (*P* < 0.001).

**Table 6:** Metastasis-free survival analysis (N = 20).

Factor	Patients	Events (metastasis)	Median (months)	*P*-value (log rank test)
**Age at diagnosis (years)**				
≤ 15	6	4	11.1	0.984
> 15	14	9	9.7	
**Gender**				
Male	11	10	8.9	**0.027**
Female	9	3	Undefined	
**Enneking stage**				
IIB	18	11	10.8	**< 0.001**
III	2	2	1.2	
**Site**				
Extremities	16	11	9.5	0.478
Axial	4	2	24.2	
**IMPDH2 expression**				
Low (IRS ≤ 6)	6	2	Undefined	**0.030**
High (IRS > 6)	14	11	6.9	
**HPRT expression**				
Low (IRS ≤ 6)	9	5	11.5	0.640
High (IRS > 6)	11	8	9.3	
**IMPDH2/HPRT ratio**				
Low IMPDH2/HPRT	16	11	9.1	0.472
High IMPDH2/HPRT	4	2	53.4	

*P* values were calculated using the log-rank test. *P* values < 0.05 shown in bold.

**Table 7:** Multivariate analysis of metastasis-free survival in osteosarcoma patients and IMPDH2 expression in post-chemotherapy tissues (n = 20).

Factor	Overall survival	
	HR (95% CI)	*P *value
**Age at diagnosis (years)**		
≤ 15	1.00	–
> 15	0.66 (0.15–2.84)	0.576
**Gender**		
Male	1.00	–
Female	0.21 (0.05–0.88)	0.033
**Site**		
Extremities	1.00	–
Axial	0.71 (0.14–3.73)	0.687
**IMPDH2 expression**		
Low (IRS ≤ 6)	1.00	–
High (IRS > 6)	7.42 (1.22–45.06)	**0.030**

**Table 8:** Multivariate analysis of metastasis-free survival in osteosarcoma patients and HPRT expression in post-chemotherapy tissues (n = 20).

Factor	Overall survival	
	HR (95% CI)	*P *value
**Age at diagnosis (years)**		
≤ 15	1.00	–
> 15	1.69 (0.47–6.11)	0.423
**Gender**		
Male	1.00	–
Female	0.24 (0.06–0.95)	0.042
**Site**		
Extremities	1.00	–
Axial	0.58 (0.12–2.96)	0.517
**HPRT expression**		
Low (IRS ≤ 6)	1.00	–
High (IRS > 6)	1.07 (0.32–3.55)	0.909

**Table 9:** Multivariate analysis of metastasis-free survival in osteosarcoma patients and IMPDH2/HPRT ratio in post-chemotherapy tissues (n = 20).

Factor	Overall survival	
	HR (95% CI)	*P *value
**Age at diagnosis (years)**		
≤ 15	1.00	–
> 15	2.22 (0.56–8.84)	0.257
**Gender**		
Male	1.00	–
Female	0.25 (0.06–1.00)	0.051
**Site**		
Extremities	1.00	–
Axial	0.45 (0.08–2.38)	0.348
**IMPDH2/HPRT ratio**		
Low IMPDH2/HPRT	1.00	–
High IMPDH2/HPRT	0.47 (0.08–2.55)	0.380

## Discussion

Unrestricted proliferation of cancer cells is limited by the abundance of total nucleotide pools as well as the level and the activity of rate-limiting enzymes of nucleotide synthesis^33^. Nucleotide biosynthesis is formed in two major ways (1) by a de novo biosynthetic pathway through biochemical reactions of simple metabolites including glucose, amino acids, glycine, CO_2_, and one-carbon units derived from the serine-glycine pathway, and (2) nucleotide salvage pathway that utilizes free bases recycled from the degradation of DNA and RNA or from exogenous dietary intake^34^.

To our knowledge, this is the first report on an association of HPRT expression with clinical outcomes and survival rate of osteosarcoma patients. Present study showed that HPRT was independent indicators of a poor prognosis, where a low HPRT level in biopsy tissues indicated shorter survival of the patient. Recent study showed that to meet high metabolic demand for cell growth, cancer cells tune the GTP biosynthesis by stimulating de novo pathway with reduced salvage pathway^34,35^. Sasaki et al*.* demonstrated that *IMPDH2* was upregulated in glioblastoma tissues, while *HPRT1* were reciprocally downregulated showing significant correlation with *IMPDH2* levels^35^. Our results also revealed that IMPDH2/HPRT ratio is an independent prognostic marker, where a high IMPDH2/HPRT ratio associated with shorter survival of the patient. However, the result from multivariate analysis revealed a significant association between HPRT levels and survival rate of osteosarcoma patients, but not for IMPDH2 expression results (Table 3). Therefore, a prognostic power of IMPDH2/HPRT ratio was, at least in part, influenced by an alteration of HPRT levels.

Until now, there have not been many studies of the nucleotide metabolic switch in osteosarcoma, but there are some indications of the importance of nucleotide metabolism, in particular, de novo guanine nucleotide synthesis. Fellenberg et al*.* showed that IMPDH2 was one of eight drug-regulated genes highly expressed in chemo-resistant osteosarcoma cells^36^. A later study also demonstrated an association between high IMPDH2 level and poor response to chemotherapy and low metastasis-free survival in osteosarcoma patients^37^. IMPDH2 expression is also an independent prognostic biomarker for other cancer types. It has been reported that IMPDH2 expression is related to advanced stage and distant metastasis in nasopharyngeal carcinoma^38^. High IMPDH2 levels also significantly indicate shorter overall survival and disease-free survival. Enhanced IMPDH2 expression is significantly associated with advanced stage, metastasis, and higher Gleason scores in cases of prostate cancer^39^.

Our findings also suggest that high levels of IMPDH2 in post-chemotherapy tissues are significantly associated with shorter metastasis-free survival. Multivariate analysis showed the independent prognostic power of IMPDH2 expression. Several reports have previously demonstrated the role of IMPDH and de novo guanine nucleotide synthesis in cancer metastasis and progression. A study by Zhou et al*.* showed that the level of IMPDH2 was enhanced in prostate cancer with metastasis^39^. Interestingly, one study demonstrated GTP-biosynthetic enzymes, including IMPDH1 and IMPDH2, were highly accumulated at the leading edge of renal carcinoma cells^40^. This plasma membrane localization of IMPDH enzymes might promote localized nucleotide metabolism which augments cancer cell migration and metastasis. Bianchi-Smiraglia et al. reported an increased level of guanosine monophosphate synthase (GMPS), an enzyme that catalyzes the amination of xylitol monophosphate (XMP) to guanosine monophosphate (GMP) in de novo guanine nucleotide synthesis pathways in metastatic human melanoma samples^41^. GMPS inhibitor could also potentially modulate melanoma cell invasion in vitro.

The main reason MPA, an IMPDH inhibitor, is a potent immunosuppressant agent is that lymphocytes are lacking salvage capability to restore level of GTP pool in the cells^30^. The present findings on an association of low HPRT level and unfavorable clinical outcomes suggest a potential metabolic vulnerability of poor prognostic patients. Currently, we are running phase II multi-center clinical trial to evaluate the efficacy and safety of mycophenolate mofetil (MMF) in patients with high-grade locally advanced or metastatic osteosarcoma (ESMMO) (Thai Clinical Trials Registry number: TCTR20190701 001)^42^. A total of 27 high-grade locally advanced or metastatic osteosarcoma patients have been recruited into the study from collaborating hospitals in Thailand.

In summary, our findings strengthen the concept that nucleotide metabolic switches and de novo guanine synthesis in tumorigenesis and osteosarcoma progression could be used as prognostic markers and to indicate actionable targets for osteosarcoma treatment.
